# Identification of *Aedes aegypti* Long Intergenic Non-coding RNAs and Their Association with *Wolbachia* and Dengue Virus Infection

**DOI:** 10.1371/journal.pntd.0005069

**Published:** 2016-10-19

**Authors:** Kayvan Etebari, Sultan Asad, Guangmei Zhang, Sassan Asgari

**Affiliations:** Australian Infectious Disease Research Centre, School of Biological Sciences, The University of Queensland, Brisbane, Australia; Colorado State University, UNITED STATES

## Abstract

Long intergenic non-coding RNAs (lincRNAs) are appearing as an important class of regulatory RNAs with a variety of biological functions. The aim of this study was to identify the lincRNA profile in the dengue vector *Aedes aegypti* and evaluate their potential role in host-pathogen interaction. The majority of previous RNA-Seq transcriptome studies in *Ae*. *aegypti* have focused on the expression pattern of annotated protein coding genes under different biological conditions. Here, we used 35 publically available RNA-Seq datasets with relatively high depth to screen the *Ae*. *aegypti* genome for lincRNA discovery. This led to the identification of 3,482 putative lincRNAs. These lincRNA genes displayed a slightly lower GC content and shorter transcript lengths compared to protein-encoding genes. *Ae*. *aegypti* lincRNAs also demonstrate low evolutionary sequence conservation even among closely related species such as *Culex quinquefasciatus* and *Anopheles gambiae*. We examined their expression in dengue virus serotype 2 (DENV-2) and *Wolbachia* infected and non-infected adult mosquitoes and Aa20 cells. The results revealed that DENV-2 infection increased the abundance of a number of host lincRNAs, from which some suppress viral replication in mosquito cells. RNAi-mediated silencing of lincRNA_1317 led to enhancement in viral replication, which possibly indicates its potential involvement in the host anti-viral defense. A number of lincRNAs were also differentially expressed in *Wolbachia*-infected mosquitoes. The results will facilitate future studies to unravel the function of lncRNAs in insects and may prove to be beneficial in developing new ways to control vectors or inhibit replication of viruses in them.

## Author Summary

*Aedes aegypti* is a major vector of several viruses such as dengue and Zika viruses. Understanding the intricate interaction of viruses with mosquito vectors and the factors involved in virus replication are essential for developing effective arbovirus control strategies. In this study, we report a comprehensive list of long intergenic non-coding RNAs encoded by the genome of *Ae*. *aegypti* for the first time. In addition, we show that a number of these long non-coding RNAs are differentially expressed in mosquitoes infected with dengue virus, which could be involved in DENV-mosquito interaction. The outcomes provide a new avenue to explore mosquito biology and mosquito-virus interactions that may lead to the discovery of molecules that could be beneficial for vector manipulation.

## Introduction

Dengue and Zika viruses are related mosquito-borne viruses that have a common primary vector, *Aedes aegypti* and infect millions of people worldwide [1,2]. Recent outbreaks of Dengue and Zika in South America pose a serious risk for other tropical regions in the world as *Ae*. *aegypti* is one of the most abundant mosquito species in these areas [2]. Although certain vaccines have been licensed in some countries, there are no efficient specific therapeutics available for either diseases, hence, the best protection against their global spreading is an efficient vector control program [3,4].

The genome sequence of *Ae*. *aegypti* is available, however, it has not been fully annotated. Only 2% of its large genome (1.376 Mb) has been annotated as protein coding genes and it reflects the presence of great proportions of non-coding transcripts as well as repetitive elements [5]. Transcriptomic changes, including those of non-coding transcripts, could provide a genome scale insight into host-pathogen interactions. Previous studies identified a series of small ncRNAs in *Ae*. *aegypti* and demonstrated their interaction with arboviruses [6–9], but our knowledge about their long ncRNAs is limited.

RNA transcripts longer than 200 nucleotides, which do not contain an open reading frame of longer than 100 amino acids, are simply defined as long ncRNA [10]. Generally, they are classified by their location relative to their neighboring protein-coding genes and include the long intergenic ncRNA (lincRNA), intronic lncRNA, antisense lncRNA and enhancer RNA [10]. Although a number of mammalian lncRNAs have been characterized and identified in the last few years, genome-wide identification of this class of ncRNAs has only recently become possible with the arrival of deep sequencing technologies. An expanding body of evidence reveals that lncRNAs, once described as dark matter, are involved in many biological processes such as genomic imprinting and cell differentiation [11]. They also play important roles in epigenetic and non-epigenetic based gene regulation [12]. Relatively, little is known about their involvement in activation and differentiation of immune cells, but new discoveries have revealed the involvement of lncRNA in defense systems [13]. Previous works have also outlined their quick responses to different stimuli and stress factors [14–17]. In addition, it has been shown that some lncRNAs enhance virus replication or decrease antiviral immunity [18].

Although in most host-virus interaction studies typically protein-coding genes have been the center of attention, there are few examples of virus and host lncRNA interactions in human and mouse models [18,19]. For instance, Hepatitis B virus (HBV) infection altered lncRNA profiles in patients, with about 4% of human lncRNAs showing more than 2-fold changes in HBV infected liver tissue [20]. Winterling *et al*. (2014) identified a virus inducible lncRNA, which is induced by vesicular stomatitis virus and several strains of Influenza A virus (IAV) [18].

The sequence and structure of lncRNAs are important in their function, in particular for their interaction with DNA, RNA, or proteins. In case of extensive base-pairing of lncRNA with target mRNA, translation can be stabilized, while partial base-pairing may accelerate mRNA decay or inhibit translation of the target mRNA [21]. It has been shown that some lncRNAs interact with other small ncRNAs such as miRNAs. For example, in silkworm, 69 lncRNAs originating from 33 gene loci, may serve as miRNA precursors, and 104 lncRNAs may function as competing endogenous RNAs (ceRNAs) [22]. LncRNAs are also targeted by miRNAs similar to mRNAs and reduce their stability. They may also act as sponge or decoy of miRNAs, and compete with miRNAs for binding to mutual target mRNAs [21].

In insects, only a few genes have been experimentally annotated as lncRNA. It has been estimated that more than 5000 loci potentially encode non-coding transcripts in *Drosophila melanogaster*, however, just seven loci (bxd, Hsrω, pgc, roX1, rox2, sphinx and yar) have been annotated as functional regulatory lncRNAs by experimentally derived data [23,24]. We recently found that a number of lncRNAs in *Plutella xylostella*, a pest of cruciferous plants, were linked to the insect’s resistance to insecticides and might be involved in detoxification processes [14]. Jenkins et al (2015) identified 2,949 lncRNAs in the malaria mosquito vector, *Anopheles gambiae*, using RNA-Seq data [25]. They showed that in various *Anopheles* species, lncRNAs have considerably lower sequence conservation as compared with protein-coding genes. In another study, it has been shown that 43% of total midgut transcripts of *An*. *gambiae* are lncRNAs and 32% of them showed some level of homology to other species [26].

The current study generated a comprehensive list of *Ae*. *aegypti* lincRNAs, which will be a complement to the other ncRNAs (microRNAs and piRNAs) that have already been discovered in this medically important species. This work also helps to improve the present annotation of the genome of *Ae*. *aegypti*. We also examined the expression pattern of some selected lincRNAs in response to microbial challenge namely dengue virus serotype 2 (DENV-2) and *Wolbachia* infections to identify potential immune-related lincRNAs in *Ae*. *aegypti* [27,28]. The results help better understanding of mosquito-pathogen interactions providing new insights on the potential role of lncRNAs as candidates for exploitation to inhibit replication of mosquito-borne viruses.

## Methods

### RNA-Seq Data preparation

Previously sequenced RNA-Seq raw data of *Ae*. *aegypti* were downloaded from NCBI Sequences Read Archive and ArrayExpress Archive with accession numbers SRA048559, SRA058076, SRA244067 and ERP002530 [29–32]. Raw data were stripped of adapters using CLC Genomic Workbench version 7.5.1 and reads with quality score of above 0.05 and maximum 2 ambiguous sequences were retained for further analysis.

### Large gap mapping and transcript discovery

The CLC Genomic workbench’s Transcript Discovery plugin was used for lincRNA discovery in the *Ae*. *aegypti* genome. New transcripts were identified by large gap mapping of 1,148,814,115 reads of 35 RNA-Seq libraries to the genomic reference (AaegL3.3). We implemented strict mapping criteria (mismatch, insertion and deletion costs: 2: 3: 3 respectively). The minimum similarity and length fraction of 0.9 between a mapped segment and the reference were allowed as part of the mapping criteria. The large gap mapper algorithm also requires each mapped segment to include at least 10% of the read with minimum length of 17 bases. We considered a gap with maximum of 50 Kbp distance between mapped read segments to span the introns from RNA-Seq data. The annotations were generated by inspecting mapping of reads and identifying likely regions corresponding to genes, including their exons and splice sites. The algorithm scans each gap in the read mapping to explore whether the gap is assigned to a valid splice site or can be relocated to a valid splice site without cost.

### lincRNA identification pipeline

**Fig 1:**
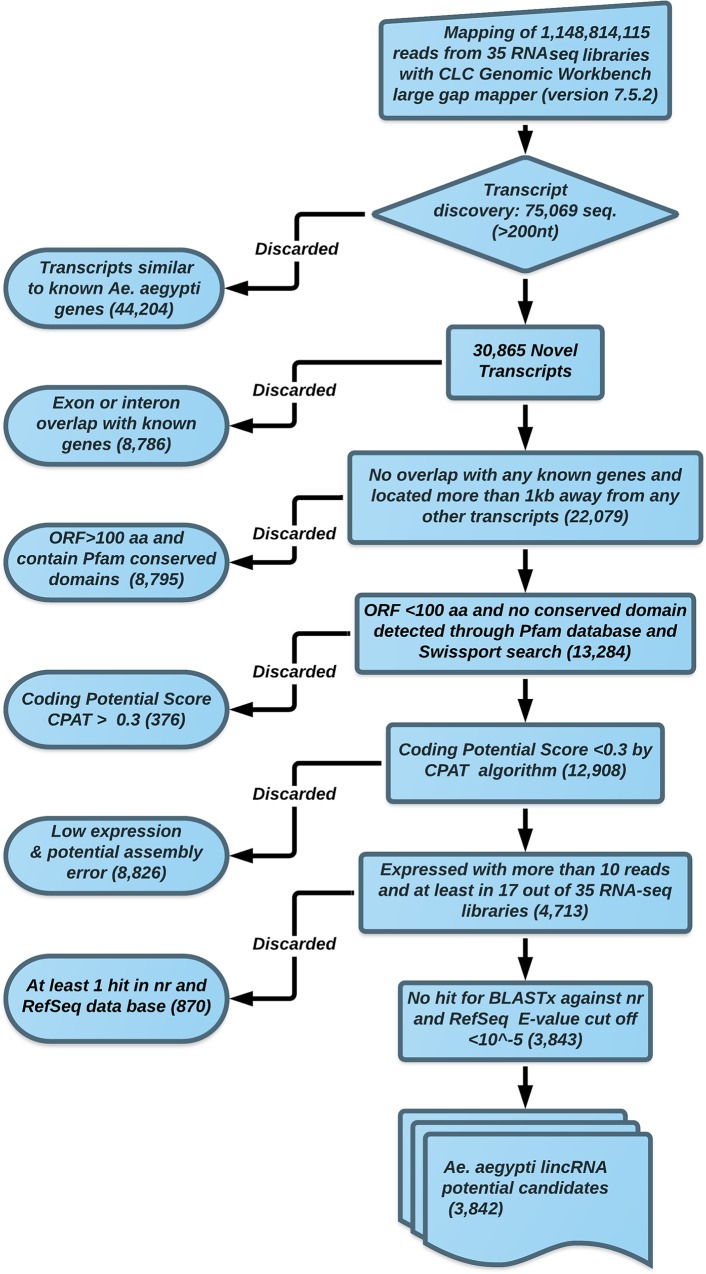
The lincRNA identification pipeline flowchart.

A rigorous filtering pipeline was developed to remove transcripts that may potentially encode proteins. The pipeline for *Ae*. *aegypti* lincRNA discovery is summarized in Fig 1. We identified 75,069 potential genes using the CLC Genomic Workbench transcript discovery algorithm. The genes that were annotated as known *Ae*. *aegypti* protein-coding genes were discarded and 30,865 potential genes were also checked for any exon or intron overlap with other known *Ae*. *aegypti* protein-coding genes. We selected 22,079 sequences, which were located more than 1kb away from any other known transcripts, for finding putative open reading frames (ORF). All possible six frames were produced for all selected sequences and then the translated sequences were subjected to a domain search to identify any putative conserved protein domains through Pfam v27.0 database [33]. We discarded 8,795 sequences with potential ORF above 100 aa or conserved protein domains. The remaining sequences were submitted to a coding potential assessment tool (CPAT), which utilizes a logistic regression model made with four sequence features: ORF size, ORF coverage, Fickett TESTCODE statistics and hexamer usage bias [34]. We applied the coding probability threshold of 0.3, which led to discarding 376 sequences as putative coding RNAs. We also implemented an expression threshold on our data to strengthen the identification pipeline. Sequences with more than 10 mappable reads in at least 17 out of 35 RNA-Seq libraries were considered as valid sequences and were kept for the next step. Any possible similarity with other known proteins was found by using BLASTx algorithm against nr and Swiss port database (E-value cut off 10^−5^). Finally, 3,842 potential lincRNAs were identified and used for further analysis.

To identify *Ae*. *aegypti* putative lincRNAs that are regarded as small RNA associated lincRNAs, we used the Blast algorithm to search for *Ae*. *aegypti* precursor miRNA sequences in the predicted *Ae*. *aegypti* lincRNA dataset. We also used publicly available small RNA libraries from DENV-infected and non-infected samples (SRP026241) in this analysis for further characterization of lincRNA_1317. All known *Ae*. *aegypti* miRNA sequences were mapped to lincRNA_1317 for possible best fitting using RNAhybrid, which is a tool for finding the normalized minimum free energy (mfe) of RNA. We did not allow G:U pairing in the seed region (nucleotide 2–8) and required miRNA-lincRNA duplexes to have a helix in this region. Maximum 5nt were allowed as unpaired nucleotides in either side of an internal loop. LncTar algorithm [35] was used to explore any potential interaction between lincRNA_1317 and DENV-2 genome (accession no. NC_001474.2) by finding the normalized mfe joint structure of two RNA molecules based on base pairing.

### Identification of differentially expressed lincRNAs upon infection

The *Ae*. *aegypti* genome was annotated with the final list of lincRNAs and used as reference for RNA-Seq analysis in CLC Genomic Workbench. To measure the lincRNA normalized expression value, RPKM (Reads Per Kilobase per Million reads) was assigned for each library [36]. To find the differential expression pattern of lincRNAs in response to DENV infection, data from DENV-2 (Jam1409) infected midgut and carcass tissues at 4 days post-infection (dpi) were compared with their corresponding control groups [30]. Baggerley's test, a count based statistical analysis was done on the data. The samples were given weights depending on their total counts. Based on the test the weights are obtained by supposing a Beta distribution on the proportions in a group, and estimating these, along with the proportion of a binomial distribution. We selected 20 potential lincRNAs with more than 4-fold change for further analysis with RT-qPCR in DENV-2 (New Guinea C strain) infected *Ae*. *aegypti* cell line (Aa20) and screened their expression profile in *Wolbachia*-infected mosquitoes.

*Ae*. *aegypti* infected with the *w*MelPop-CLA strain of *Wolbachia* (+Wol) and without *Wolbachia* (-Wol, tetracycline-cured line) were stocks produced previously [37]. For the experiments in this work, 4-day-old female mosquitoes were used from which total RNA was extracted with 6–10 adult mosquitoes for each biological replicates.

### Expression analysis of *Ae*. *aegypti* lincRNAs

Detection and validation of the relative abundance of selected lincRNAs was carried through lincRNAs’ specific primers using SYBR Green chemistry in real time PCR machine. Briefly, total RNA was extracted from cells using Qiazol reagent according to the manufacturer’s instructions (Qiagen). The TURBO DNA-free kit (Ambion, USA) was used to remove possible genomic DNA contamination in RNA samples. First strand cDNA was synthesized from 2.5 μg of RNA using a poly-dT primer and Superscript III reverse transcriptase (Life Technologies). qPCR primers were designed using primer design tool of NCBI [38]. QuantiFast SYBR Green PCR Master Mix with ROX was used to quantify the relative expression of lincRNAs between different treatments. Three independent biological replicates were considered along with three technical replicates for each treatment. Reactions were performed in a Rotor-Gene thermal cycler (Qiagen) under the following conditions: 95°C for 5 min, and 40 cycles of 95°C for 10s and 60°C for 30s, followed by the melting curve (68°C to 95°C). Melting curves were analysed to examine the specificity of amplification. Relative expressions were calculated using the Rotor-Gene software and the mosquito *RPS17* as reference gene for normalization. Unpaired t-test was used to identify statistically significant differences.

### RNAi of selected lincRNAs and virus replication assay

To check the functional importance of the identified novel lincRNAs, dsRNAs were synthesized to knockdown selected lincRNAs (2329, 1613 and 1317) to check their effect on DENV replication. Briefly, primers with added T7 promoter sequence (S1 **Table**) were used to generate 250–600 bp PCR products from selected lincRNAs. Megascript T7 kit (Ambion) was used according to the manufacturer’s instruction to generate respective dsRNAs. To induce efficient RNA silencing, *Ae*. *aegypti* Aa20 cells were double transfected with dsRNAs against selected lincRNAs. Aa20 cells were re-suspended and ~3×10^5^ cells were added to each well of a 12-well plate. Cells were allowed to settle for ~1 h, medium was removed and replaced with a transfection mixture consisting of 0.5 ml medium (1:1 Schneider medium and Mitsuhashi–Maramorosch with 10% FBS), 8 μl Cellfectin (Invitrogen), and 5 μg dsRNA either for selected lincRNAs or GFP as control. Cells were also treated with 3 μg dsRNA 72 h after the primary transfection to increase the silencing efficiency of selected lincRNAs. Six hours after the secondary transfection, cells were infected at 1 multiplicity of infection (MOI) with DENV-2 (New Guinea C strain). All the treatments were collected three days post-infection. RNA extraction and cDNA synthesis were carried out as above. qPCR was performed to confirm the knockdown and the effect of particular lincRNA knockdown on the genomic RNA of DENV-2. Each treatment was repeated three times. All data from three biological replicates were subjected to one-way ANOVA statistical analysis. Brown-Forsythe test was used to check the equality of group variances and Tukey's multiple comparisons test was also used to examine significant statistical differences among treatments.

## Results and Discussion

### Identification and characterisation of *Ae*. *aegypti* lincRNAs

**Fig 2:**
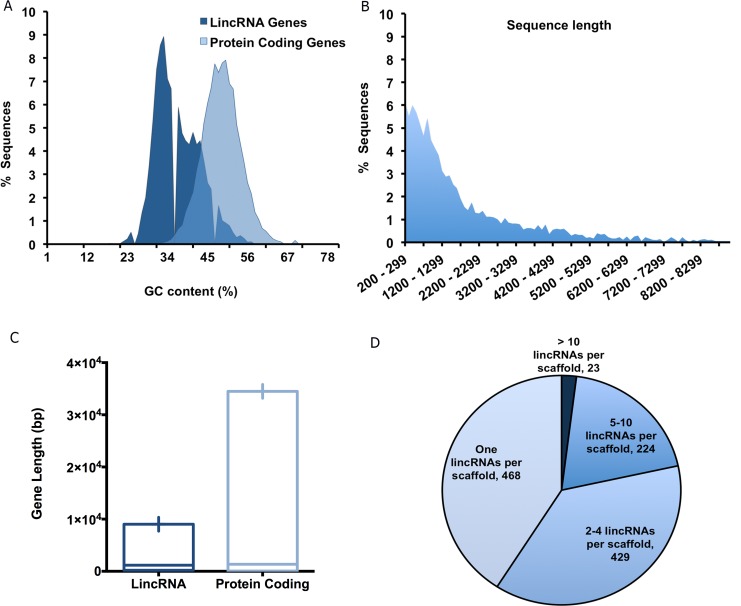
*Aedes aegypti* lincRNA characterization. A) Comparison of the GC content in protein-coding genes and the putative lincRNA genes. B) Sequence length distribution of *Ae*. *aegypti* lincRNA candidates. C) Comparison of gene length in protein-coding genes and putative lincRNA genes. D) lincRNA distribution among different *Ae*. *aegypti* genome scaffolds. The majority of scaffolds (~77%) only contain 1–4 lincRNAs, while only 23 *Ae*. *aegypti* genome scaffolds contain more than 10 lincRNAs (~2%).

In total, 3,482 putative lincRNAs in 1,114 *Ae*. *aegypti* genome scaffolds were identified (S2 Table). The *Ae*. *aegypti* lincRNA genes displayed a slightly lower GC content (mean: 40.1%) in comparison to 47.8% in their protein-coding gene sequences (Fig 2A). The lower GC content or AT enrichment is a typical characteristic of lincRNAs and our findings are congruent with predicted lincRNAs in other species [14,39,40]. The majority of *Ae*. *aegypti* predicted lincRNAs are smaller than 3000 bases and their length distribution is represented in Fig 2B. These mosquito lincRNA candidates are notably shorter in length than protein-coding genes, demonstrating another well-known characteristic of lincRNA transcripts (Fig 2C) [41,42]. The majority of *Ae*. *aegypti* genome scaffolds contain less than five lincRNA loci (~80%), however, 23 of scaffolds (2%) were enriched with more than 10 lincRNAs (Fig 2D). The detailed information of these scaffolds, which contain the highest number of lincRNAs are summarized in Table 1.

**Table 1:** Distribution of potential *Ae*. *aegypti* lincRNAs in different genome scaffolds with more than 10 lincRNAs and their comparison with the number of protein-coding genes.

Scaffold	Length (kbp)	Number of known genes	Number of lincRNAs	Length range (bp)	Average size of lincRNA
supercont1.1	5,856,339	124	20	261–7780	1,869
supercont1.16	4,402,401	62	19	264–7065	1,761
supercont1.35	3,598,302	34	17	420–6101	1,850
supercont1.3	5,167,134	61	16	297–5683	1,897
supercont1.19	4,221,289	48	14	339–6537	1,776
supercont1.70	2,929,944	29	14	509–6314	2,039
supercont1.37	3,744,586	35	13	497–8297	3,585
supercont1.28	3,768,427	45	13	235–7876	2,229
supercont1.29	3,855,786	42	13	301–5559	2,376
supercont1.78	2,909,025	17	13	211–5561	1,497
supercont1.18	4,266,046	76	12	400–7336	2,087
supercont1.6	5,075,626	93	12	547–3182	1,380
supercont1.225	1,723,990	19	12	489–4486	1,475
supercont1.38	3,498,553	45	12	248–8797	2,080
supercont1.46	3,321,798	42	12	248–7447	2,210
supercont1.5	5,058,281	60	12	323–4291	2,168
supercont1.120	2,427,180	38	11	805–5190	2,116
supercont1.244	1,610,334	30	11	503–6058	2,512
supercont1.49	3,164,279	51	11	324–5492	1,988
supercont1.107	2,543,601	42	11	416–8896	2,876
supercont1.22	4,100,794	51	11	409–4234	1,441
supercont1.44	3,232,429	47	11	271–8994	2,175
supercont1.92	2,802,290	34	11	242–4424	1,667

We examined all the identified lincRNAs to determine their association with *Ae*. *aegypti* miRNA precursors and piRNA clusters. We found that the pre-miRNA sequences of aae-miR-2940 and aae-miR-285 are located in lincRNAs 1431 and 3299, respectively. We could not detect any other pre-miRNA sequences identified in *Ae*. *aegypti* in the lincRNAs. Also, lincRNA 1978 and 792 are originated from two previously reported piRNA clusters [43] located at supercontig 1.478 and 1.98, respectively.

**Fig 3:**
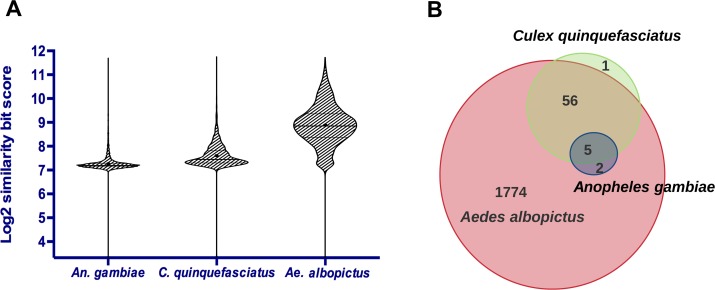
*Ae*. *aegypti* lincRNAs share some conserved areas with other closely related species. A) The similarity bit score showed more similar sequences with high-degree of similarity were present in *Ae*. *albopictus*. B) The Venn diagram displays the number of *Ae*. *aegypti* lincRNAs with similarity scores above the cut off (E-value above 10^−50^) in other species using the BLAST algorithm.

LincRNAs demonstrate low evolutionary sequence conservation even among closely related species [10,14]. We used the BLAST algorithm bit score to identify the level of similarity among *Ae*. *aegypti* lincRNA sequences with other closely related insect genomes such as *Aedes albopictus*, *Culex quinquefasciatus* and *Anopheles gambiae* (Fig 3A). As expected, most of the identified lincRNAs showed high level of similarity with *Ae*. *albopictus* genome sequence and probably are genus specific. The E-value cut off 10^−50^ was applied to our screening with the BLAST algorithm to identify the conserved sequences. Although the *Ae*. *aegypti* lincRNAs shared high level of sequence similarity with the genome of *Ae*. *albopictus*, only 62 and 7 lincRNAs had sequence similarity with *Cx. quinquefasciatus* and *An*. *gambiae*, respectively (Fig 3B). They were mostly limited to a single short region with high conservation.

### *Ae*. *aegypti* lincRNAs change upon microbial challenge

**Fig 4:**
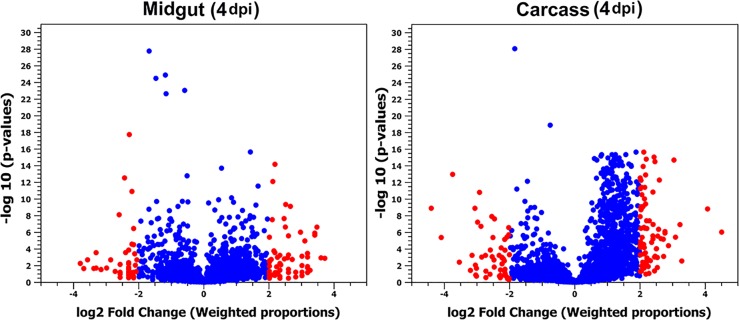
Volcano plot of differentially expressed *Ae*. *aegypti* lincRNAs in DENV-2 infected samples (midgut and carcass) compared with their corresponding controls. Dots with red color represent lincRNAs with more than 4-fold changes due to DENV-2 infection.

Following the identification of *Ae*. *aegypti* lincRNAs, we analyzed their transcript levels in DENV-2 infected mosquitoes. To produce the lincRNA profile of infected and non-infected mosquitoes, we re-analyzed previously published RNA-Seq data from *Ae*. *aegypti* midgut and carcass samples at 4 dpi [30] (Fig 4). 248 and 203 lincRNAs with fold changes above four were identified in the RNA-Seq libraries of midgut and carcass, respectively (S3 Table). The majority of differentially expressed lincRNAs were considerably overexpressed in both samples. The abundance of only 32% of *Ae*. *aegypti* lincRNA candidates decreased in response to DENV-2 infection in the mosquito carcass sample. Thirty lincRNAs were differentially expressed in both examined samples. The transcription levels of 72 lincRNAs increased after infection while their expression could not be detected in the non-infected midgut tissue sample.

**Fig 5:**
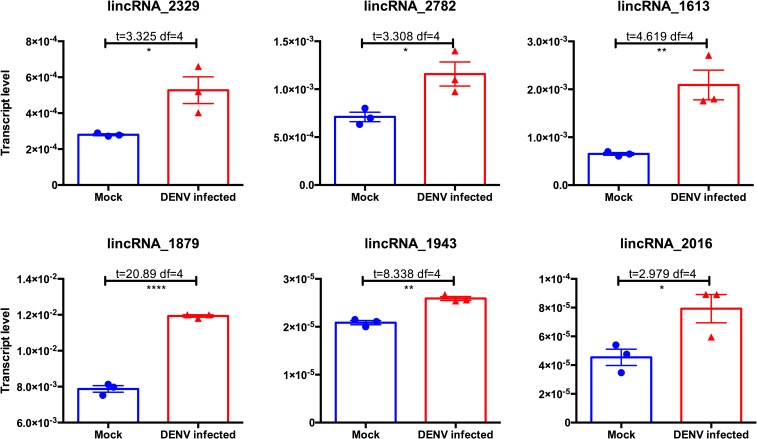
DENV infection leads to changes in the abundance of *Ae*. *aegypti* lincRNAs. The relative transcript levels of selected numbers of *Ae*. *aegypti* lincRNAs were measured by RT-qPCR analysis of Aa20 cells infected with 1 MOI of DENV-2 for three days. Three biological replicates were used for each treatment with three technical replicates each. *, p < 0.05; **, p < 0.01; ****, p < 0.0001.

We selected 20 candidates of those differentially expressed lincRNAs from RNA-Seq analysis data for further investigation. The relative expression of lincRNA candidates were examined by reverse transcription quantitative polymerase chain reaction (RT-qPCR) upon DENV-2 infection in Aa20 mosquito cells. Only significantly overexpressed lincRNAs after DENV-2 infection are represented in Fig 5. Although we used Aa20 cells for the lincRNA expression assays, the expression patterns of almost all the examined lincRNAs (5 out of 6) were consistent with the RNA-Seq data (adult mosquito carcass sample). We used a poly-dT primer to produce cDNA, which also confirmed that all of those identified transcripts have poly-A tails and therefore are true transcripts. Based on these results, significant increase in the transcription levels of a selected number of *Ae*. *aegypti* lincRNAs suggests their possible involvement in host-pathogen interaction but further investigations are required to confirm their roles in antiviral/immune responses.

**Fig 6:**
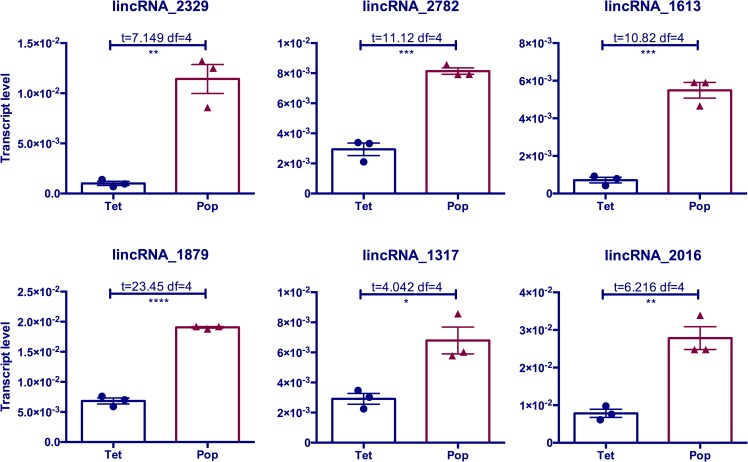
The transcript levels of *Ae*. *aegypti* lincRNAs were altered in *Wolbachia*-infected mosquitoes. RT-qPCR was used to analyze the relative transcript levels of selected numbers of *Ae*. *aegypti* lincRNAs in response to *Wolbachia* infection. For this, RNA from 4-day-old female mosquitoes from *w*MelPop (Pop)-infected and their tetracycline-cured line (Tet) mosquitoes were used in three biological replicates, each with three technical replicates. *, p < 0.05; **, p < 0.01; ***, p < 0.001; ****, p < 0.0001.

We also examined the impact of an endosymbiotic bacterium, *Wolbachia*, on some selected *Ae*. *aegypti* lincRNAs, which showed significant changes in response to DENV-2 infection. This gram-negative bacterium is transmitted maternally and potentially infects more than 40% of all insect species, manipulating its hosts using different strategies [44,45]. A fascinating aspect of *Wolbachia* infection is limiting replication of vector-borne pathogens in mosquitoes [45,46]. However, the mechanism(s) behind virus blocking is largely unknown. Here, we found that the transcript levels of several lincRNA genes significantly increased in *Wolbachia*-infected *Ae*. *aegypti* mosquitoes (Fig 6), which may lead to differential regulation of cellular protein-coding genes. Our previous studies showed that *Wolbachia* could manipulate host small ncRNAs such as miRNAs and piRNAs [47]. An overall induction of small ncRNAs between 18 and 28 nucleotides was also observed in *Ae*. *aegypti* cell line infected with *w*MelPop-CLA strain of *Wolbachia* [48]. It was assumed that the upregulation of small ncRNAs in infected cells may result in an enhanced immune response and activated RNAi pathway. However, the role of these modifications in the host lincRNA gene expression profile, and potentially in anti-viral responses, is unknown and may lead to the discovery of lincRNAs that could be utilized for inhibition of virus replication in mosquitoes.

A recent study on mouse bone marrow-derived macrophage (BMDM) model reported a significant upregulation in 72 lincRNAs after treatment with the synthetic bacterial lipoprotein Pam3CSK4, which acts through Toll-like receptor [49]. In another study, differential expression of approximately 500 annotated mouse lncRNAs was reported during infection with severe acute respiratory syndrome coronavirus [50]. Recently, it has been shown that honeybee lincRNAs are also differentially expressed during infection with various viruses such as sacbrood virus (SBV) and deformed wing virus (DWV), but the biological significance of these lincRNAs is completely unknown [51]. Although exploring the *in vivo* functions of immune-related lincRNAs is one exciting area for future studies, the differential expression of some lincRNAs could simply be byproducts of mRNA biogenesis or changes in global transcriptional profile due to microbial challenges [52,53]. Struhl (2007) believed that the transcriptional machinery is not perfect producing RNAs that serve no purpose or have no significant role in infection [54]. On the other hand, there are several examples which have shown that lincRNAs could be potentially important factors in host antimicrobial responses, and may represent a new class of signaling molecules involved in innate immunity or provide a new layer in gene regulation. For instance, two interferon (INF) induced lncRNAs, which were upregulated by influenza and vesicular stomatitis viruses, regulate the expression of the antiviral factor tetherin in human HuH7 cells [55].

### RNAi of selected lincRNAs and enhancement of DENV-2 replication

**Fig 7:**
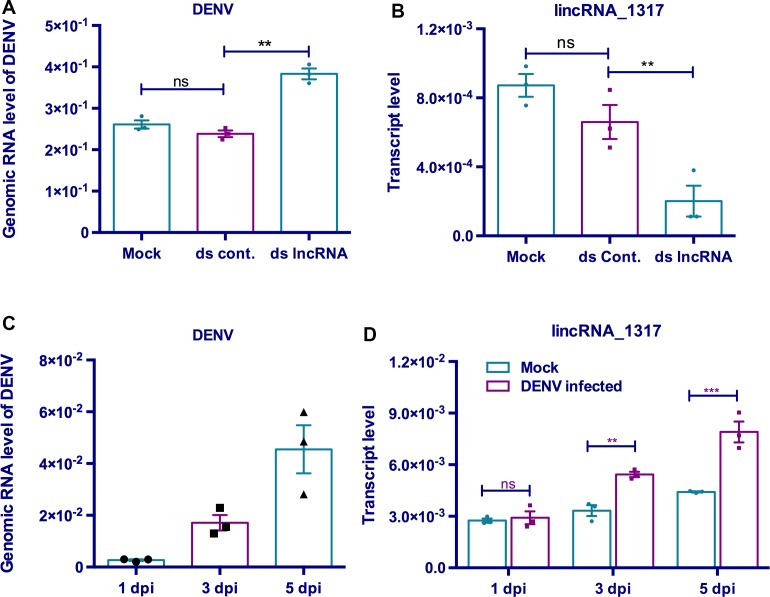
Possible involvement of lincRNA_1317 in DENV-2 replication in *Ae*. *aegypti* Aa20 cells. A) Aa20 cells were double transfected with the transfection reagent only (Mock), dsRNA to GFP (dsCont) or dsRNA to lincRNA_1317 (dslncRNA) for three days followed by 1 MOI infection of the cells with DENV-2. Primers to the NS2A region (S1 Table) were used for measuring the relative DENV-2 genomic RNA levels. B) RNAi silencing of lincRNA_1317 using dsRNA was confirmed by RT-qPCR. C) Changes in DENV-2 genomic RNA levels during the course of infection analyzed by RT-qPCR on RNA extracted from Aa20 cells at 1, 3 and 5 days post-infection (dpi). D) DENV-2 infection increased the transcript levels of *Ae*. *aegypti* lincRNA_1317 in 3^rd^ and 5^th^ days post-infection when RNA from cells were analyzed by RT-qPCR. In all the experiments shown in this figure, three biological replicates, each with three technical replicates were used. **, p < 0.01; ***, p < 0.001.

To confirm the role of DENV-induced lincRNAs in viral replication, we used RNAi-mediated silencing of two selected lincRNAs (lincRNA_1317 and 1613) using dsRNA in Aa20 cells followed by DENV-2 infection. Only RNAi-mediated silencing of lincRNA_1317 led to enhancement of DENV-2 replication (Fig 7A). Silencing of the lincRNA was confirmed by RT-qPCR (Fig 7B). Interestingly, expression of *Ae*. *aegypti* lincRNA_1317 increased substantially following the progression of infection (Fig 7C) suggesting that this lincRNA might be involved in antiviral response. This idea is consistent with the finding that lincRNA_1317 was also highly overexpressed (2.33 fold) in *Wolbachia*-infected mosquitoes as compared with non-infected mosquitoes (Fig 6).

While there are no reports on the involvement of lncRNAs in host-pathogen interactions in insects, time-dependent over-expression of host lincRNAs in response to viral infection has been observed in humans. A recent study showed more than 80% of host cell lncRNAs were upregulated upon an adenovirus infection of human primary lung fibroblast cells [56]. Zhang et al. (2013) reported alterations of expression of cellular lncRNAs in HIV-1-infected T cells. Among differentially expressed lncRNAs, NEAT1 expression notably increased in infected cells. When NEAT1 was silenced, virus production was enhanced by increasing the nucleus-to-cytoplasm export of HIV-1 transcripts containing Rev-dependent instability element [57]. A significant induction in this lncRNA expression in response to influenza virus and herpes simplex virus infection has also been shown [58].

To further investigate the potential role of lincRNA_1317 in mosquito-pathogen interaction, we determined its association with host endogenous small RNAs and its possible direct interaction with DENV. Although this lincRNA is not located in any of the known piRNA clusters, the majority of mappable small RNA reads to its sequence are in the range of 26–29 nt (S1 Fig). However, there was no difference in the mapping pattern and mapped read length distribution when reads from DENV-infected and non-infected small RNA libraries were mapped to lincRNA_1317 (S1 Fig). It has been shown that piRNA-like small RNAs have a large impact on lincRNA transcriptome [57], but our knowledge about the function of piRNA-mediated lncRNAs is still limited. Recently, it has been reported that piRNAs derived from transposons and pseudogenes facilitate the degradation of lncRNAs in mouse late spermatocytes [57].

Next, we hypothesized that *Ae*. *aegypti* lincRNA_1317 response to microbial challenge could be due to cross-regulation between miRNAs and the lincRNA. *Ae*. *aegypti* miRNA recognition elements on lincRNA_1317 were identified by calculating the normalized minimum free energy (mfe) of hybridization for each *Ae*. *aegypti* miRNA and lincRNA_1317 using RNAhybrid core script. Binding site enrichment was detected for a few miRNAs with more than two recognition elements (Table 2). For instance, more than four recognition sites were predicted for miR-278-5p and miR-252-3p on lincRNA_1317. We also identified some hot spots for miRNA recognition sites on lincRNA_1317, which may allow multiple miRNAs to bind to the same regions (S2 Fig). miRNAs can reduce lincRNA stability by targeting their transcripts similar to mRNAs. Also, lincRNAs with multiple recognition sites may actually be competitive inhibitors of miRNA function and stopping them from binding to their genuine targets by sequestering them [21]. Although the mfe for some of those miRNA-lincRNA recognition sites suggests high probability of a binding event, further experimental investigations are required to validate this interface.

**Table 2:** *Ae*. *aegypti* miRNA recognition site distribution on lincRNA_1317.

miRNA	No. recognition sites	Mean MFE (Kcal/Mol)	Recognition site start position on lincRNA_1317
miR-278-5p	5	-22.40	307, 749, 1112, 1260, 1491
miR-252-3p	4	-21.15	162, 629, 1560, 3946
miR-11-5p	3	-21.67	2248, 2286, 3328
miR-1890	3	-21.17	1489, 2712, 3602
miR-263a-3p	3	-21.47	2208, 2545, 3336
miR-33	3	-24.90	1545, 1810, 2669
miR-34-5p	3	-25.03	1020, 1232, 1379
miR-9b	3	-24.03	2034, 2891, 3603
let-7	2	-22.20	2747, 2817
miR-1	2	-22.80	1165, 2528
miR-1175-3p	2	-20.65	1489, 3274
miR-12-5p	2	-25.60	632, 1575
miR-1889-3p	2	-20.80	1268, 3988
miR-1891	2	-21.10	162, 2349
miR-282-5p	2	-25.70	1232, 1297
miR-2944b-3p	2	-22.05	1255, 3797
miR-2945-5p	2	-23.00	770, 1209
miR-31	2	-25.70	819, 871
miR-375	2	-22.25	66, 3293
miR-92b-5p	2	-23.05	1042, 3788
miR-9a	2	-20.65	170, 3500

We also used LncTar algorithm to predict any direct interaction between lincRNA_1317 and DENV-2 genome. One potential interaction was predicted in the region 1–3370 of lincRNA_1317 and the region of 3210–6579 of DENV-2 genome with mfe of -61.73 (normalized dG -0.0184). This tool has accuracy rate of 80% [35], but does not consider the tertiary structure of RNA, which could play a role in RNA–RNA interactions, and therefore further studies are required to validate any potential interaction.

The involvement of lincRNA_1317 in host response to viral infection might be through its interactions with regulatory proteins that are involved in epigenetic changes by directly interacting with chromatin modifying enzymes or DNA binding proteins such as transcription factors. This interaction has been shown in several examples in mammalian systems, including host-virus interactions in which lncRNAs mediate antiviral responses by controlling the expression of immune-related genes (reviewed in [58]).

Although our knowledge of the biological function of this class of ncRNAs in mosquitoes is still limited, the results generated from this study will facilitate forthcoming explorations of lincRNA functions in insects. Clearly, further research is required to provide concrete experimental evidence to support the role of lincRNA_1317 or any other *Ae*. *aegypti* lincRNAs in host-pathogen interaction. With advances in technology, the mosquito lincRNA-protein interactions can be identified using high-throughput sequencing of immunoprecipitated RNA after cross-linking (CLIP-Seq). Further, functional studies could be carried out to characterize immune-related lincRNAs. The involvement of lincRNAs in pathways associated with responses to viral infection and cellular stress makes them interesting candidates as potential targets for manipulation to inhibit virus replication or control vector populations.

## Supporting Information

S1 Fig Length distribution of small RNA reads mapped to lincRNA_1317. (TIF)

S2 Fig The miRNA recognition hot spot sites on lincRNA_1317. (TIF)

S1 Table List of primers used in this study. (DOCX)

S2 Table Identified lincRNA candidates in *Ae*. *aegypti* and their genome coordinates. (XLSX)

S3 Table Differentially expressed lincRNAs in response to DENV-2 infection in midgut and carcass. (XLSX)

## References

[1] Bhatt S, Gething PW, Brady OJ, Messina JP, Farlow AW, et al (2013) The global distribution and burden of dengue. Nature 496: 504–507. 10.1038/nature12060 23563266 PMC3651993

[2] Yakob L, Walker T (2016) Zika virus outbreak in the Americas: the need for novel mosquito control methods. Lancet Global Health 4: E148–E149. 10.1016/S2214-109X(16)00048-6 26848089

[3] Hennessey M, Fischer M, Staples JE (2016) Zika virus spreads to new areas—region of the Americas, May 2015-January 2016. Am J Transplant 16: 1031–1034. 10.15585/mmwr.mm6503e1 26820163

[4] Murray CL, Jones CT, Rice CM (2008) Architects of assembly: roles of Flaviviridae non-structural proteins in virion morphogenesis. Nat Rev Microbiol 6: 699–708. 10.1038/nrmicro1928 18587411 PMC2764292

[5] Nene V, Wortman JR, Lawson D, Haas B, Kodira C, et al (2007) Genome sequence of *Aedes aegypti*, a major arbovirus vector. Science 316: 1718–1723. 10.1126/science.1138878 17510324 PMC2868357

[6] Vodovar N, Bronkhorst AW, van Cleef KWR, Miesen P, Blanc H, et al (2012) Arbovirus-derived piRNAs exhibit a ping-pong signature in mosquito cells. PLoS One 7: e30861 10.1371/journal.pone.0030861 22292064 PMC3265520

[7] Campbell CL, Harrison T, Hess AM, Ebel GD (2014) MicroRNA levels are modulated in *Aedes aegypti* after exposure to Dengue-2. Insect Mol Biol 23: 132–139. 10.1111/imb.12070 24237456 PMC4120961

[8] Hess AM, Prasad AN, Ptitsyn A, Ebel GD, Olson KE, et al (2011) Small RNA profiling of Dengue virus-mosquito interactions implicates the PIWI RNA pathway in anti-viral defense. BMC Microbiol 11: 45 10.1186/1471-2180-11-45 21356105 PMC3060848

[9] Hussain M, Torres S, Schnettler E, Funk A, Grundhoff A, et al (2012) West Nile virus encodes a microRNA-like small RNA in the 3' untranslated region which up-regulates GATA4 mRNA and facilitates virus replication in mosquito cells. Nucleic Acids Res 40: 2210–2223. 10.1093/nar/gkr848 22080551 PMC3300009

[10] Clark MB, Mattick JS (2011) Long noncoding RNAs in cell biology. Semin Cell Dev Biol 22: 366–376. 10.1016/j.semcdb.2011.01.001 21256239

[11] Bonasio R, Shiekhattar R (2014) Regulation of transcription by long noncoding RNAs. Annu Rev Genet 48: 433–455. 10.1146/annurev-genet-120213-092323 25251851 PMC4285387

[12] Mercer TR, Dinger ME, Mattick JS (2009) Long non-coding RNAs: insights into functions. Nat Rev Genet 10: 155–159. 10.1038/nrg2521 19188922

[13] Fitzgerald KA, Caffrey DR (2014) Long noncoding RNAs in innate and adaptive immunity. Curr Opin Immunol 26: 140–146. 10.1016/j.coi.2013.12.001 24556411 PMC3932021

[14] Etebari K, Furlong MJ, Asgari S (2015) Genome wide discovery of long intergenic non-coding RNAs in Diamondback moth (*Plutella xylostella)* and their expression in insecticide resistant strains. Sci Rep 5: 14642 10.1038/srep14642 26411386 PMC4585956

[15] Mizutani R, Wakamatsu A, Tanaka N, Yoshida H, Tochigi N, et al (2012) Identification and characterization of novel genotoxic stress-inducible nuclear long noncoding RNAs in mammalian cells. PLoS One 7: e34949 10.1371/journal.pone.0034949 22532836 PMC3330809

[16] Tani H, Onuma Y, Ito Y, Torimura M (2014) Long non-coding RNAs as surrogate indicators for chemical stress responses in human-induced pluripotent stem cells. PLoS One 9: e106282 10.1371/journal.pone.0106282 25171338 PMC4149554

[17] Lakhotia SC (2012) Long non-coding RNAs coordinate cellular responses to stress. RNA 3: 779–796. 10.1002/wrna.1135 22976942

[18] Winterling C, Koch M, Koeppel M, Garcia-Alcalde F, Karlas A, et al (2014) Evidence for a crucial role of a host non-coding RNA in influenza A virus replication. RNA Biol 11: 66–75. 10.4161/rna.27504 24440876 PMC3929426

[19] Collier SP, Collins PL, Williams CL, Boothby MR, Aune TM (2012) Influence of Tmevpg1, a long intergenic noncoding RNA, on the expression of Ifng by Th1 Cells. J Immunol 189: 2084–2088. 10.4049/jimmunol.1200774 22851706 PMC3424368

[20] Pan Y-f, Qin T, Feng L, Yu Z-j (2013) Expression profile of altered long non-coding RNAs in patients with HBV-associated hepatocellular carcinoma. J Huazhong Univ Sci Technolog Med Sci 33: 96–101. 10.1007/s11596-013-1078-y 23392715

[21] Yoon J-H, Abdelmohsen K, Gorospe M (2014) Functional interactions among microRNAs and long noncoding RNAs. Semin Cell Dev Biol 34: 9–14. 10.1016/j.semcdb.2014.05.015 24965208 PMC4163095

[22] Wu Y, Cheng T, Liu C, Liu D, Zhang Q, et al (2016) Systematic identification and characterization of long non-coding RNAs in the silkworm, *Bombyx mori*. PLoS One 11: e0147147 10.1371/journal.pone.0147147 26771876 PMC4714849

[23] Legeai F, Derrien T (2015) Identification of long non-coding RNAs in insects genomes. Curr Opin Insect Sci 7: 37–44. 10.1016/j.cois.2015.01.003 32846672

[24] Marco A (2012) Regulatory RNAs in the light of *Drosophila* genomics. Brief Funct Genomics 11: 356–365. 10.1093/bfgp/els033 22956639 PMC4007103

[25] Jenkins AM, Waterhouse RM, Muskavitch MAT (2015) Long non-coding RNA discovery across the genus *Anopheles* reveals conserved secondary structures within and beyond the *gambiae* complex. BMC Genomics 16: 337 10.1186/s12864-015-1507-3 25903279 PMC4409983

[26] Padron A, Molina-Cruz A, Quinones M, Ribeiro JMC, Ramphul U, et al (2014) In depth annotation of the *Anopheles gambiae* mosquito midgut transcriptome. BMC Genomics 15: 636 10.1186/1471-2164-15-636 25073905 PMC4131051

[27] Lambrechts L, Ferguson NM, Harris E, Holmes EC, McGraw EA, et al (2015) Assessing the epidemiological effect of *Wolbachia* for dengue control. Lancet Infect Dis 15: 862–866. 10.1016/S1473-3099(15)00091-2 26051887 PMC4824166

[28] Moreira LA, Iturbe-Ormaetxe I, Jeffery JA, Lu G, Pyke AT, et al (2009) A *Wolbachia* symbiont in *Aedes aegypti* limits infection with Dengue, Chikungunya, and *Plasmodium*. Cell 139: 1268–1278. 10.1016/j.cell.2009.11.042 20064373

[29] David J-P, Faucon F, Chandor-Proust A, Poupardin R, Riaz MA, et al (2014) Comparative analysis of response to selection with three insecticides in the dengue mosquito *Aedes aegypti using* mRNA sequencing. BMC Genomics 15: 174 10.1186/1471-2164-15-174 24593293 PMC4029067

[30] Bonizzoni M, Dunn WA, Campbell CL, Olson KE, Marinotti O, et al (2012) Complex modulation of the *Aedes aegypti* transcriptome in response to dengue virus infection. PLoS One 7: e050512. 10.1371/journal.pone.0050512 PMC3507784 23209765

[31] Akbari OS, Antoshechkin I, Amrhein H, Williams B, Diloreto R, et al (2013) The Developmental transcriptome of the mosquito *Aedes aegypti*, an invasive species and major arbovirus vector. G3 3: 1493–1509. 10.1534/g3.113.006742 23833213 PMC3755910

[32] Chandler JA, Thongsripong P, Green A, Kittayapong P, Wilcox BA, et al (2014) Metagenomic shotgun sequencing of a Bunyavirus in wild-caught *Aedes aegypti* from Thailand informs the evolutionary and genomic history of the Phleboviruses. Virology 464: 312–319. 10.1016/j.virol.2014.06.036 25108381 PMC4157124

[33] Punta M, Coggill PC, Eberhardt RY, Mistry J, Tate J, et al (2012) The Pfam protein families database. Nucleic Acids Res 40: D290–D301. 10.1093/nar/gkr1065 22127870 PMC3245129

[34] Wang L, Park HJ, Dasari S, Wang S, Kocher J-P, et al (2013) CPAT: Coding-Potential Assessment Tool using an alignment-free logistic regression model. Nucleic Acids Res 41: e74 10.1093/nar/gkt006 23335781 PMC3616698

[35] Li J, Ma W, Zeng P, Wang J, Geng B, et al (2015) LncTar: a tool for predicting the RNA targets of long noncoding RNAs. Brief Bioinform 16: 806–812. 10.1093/bib/bbu048 25524864

[36] Mortazavi A, Williams B, McCue K, Schaeffer L, Wold B (2008) Mapping and quantifying mammalian transcriptomes by RNA-Seq. Nat Methods 5: 621–628. 10.1038/nmeth.1226 18516045 PMC13303166

[37] McMeniman CJ, Lane RV, Cass BN, Fong AWC, Sidhu M, et al (2009) Stable introduction of a life-shortening *Wolbachia* infection into the mosquito *Aedes aegypti*. Science 323: 141–144. 10.1126/science.1165326 19119237

[38] Ye J, Coulouris G, Zaretskaya I, Cutcutache I, Rozen S, et al (2012) Primer-BLAST: A tool to design target-specific primers for polymerase chain reaction. BMC Bioinformatics 13: 134 10.1186/1471-2105-13-134 22708584 PMC3412702

[39] Broadbent KM, Park D, Wolf AR, Van Tyne D, Sims JS, et al (2011) A global transcriptional analysis of *Plasmodium falciparum* malaria reveals a novel family of telomere-associated lncRNAs. Genome Biol 12: R56 10.1186/gb-2011-12-6-r56 21689454 PMC3218844

[40] Clark MB, Johnston RL, Inostroza-Ponta M, Fox AH, Fortini E, et al (2012) Genome-wide analysis of long noncoding RNA stability. Genome Res 22: 885–898. 10.1101/gr.131037.111 22406755 PMC3337434

[41] Billerey C, Boussaha M, Esquerre D, Rebours E, Djari A, et al (2014) Identification of large intergenic non-coding RNAs in bovine muscle using next-generation transcriptomic sequencing. BMC Genomics 15: 499 10.1186/1471-2164-15-499 24948191 PMC4073507

[42] Cabili MN, Trapnell C, Goff L, Koziol M, Tazon-Vega B, et al (2011) Integrative annotation of human large intergenic noncoding RNAs reveals global properties and specific subclasses. Genes Dev 25: 1915–1927. 10.1101/gad.17446611 21890647 PMC3185964

[43] Arensburger P, Hice RH, Wright JA, Craig NL, Atkinson PW (2011) The mosquito *Aedes aegypti* has a large genome size and high transposable element load but contains a low proportion of transposon-specific piRNAs. BMC Genomics 12: 606 10.1186/1471-2164-12-606 22171608 PMC3259105

[44] Zug R, Hammerstein P (2012) Still a host of hosts for *Wolbachia*: Analysis of recent data suggests that 40% of terrestrial Arthropod species are infected. PLoS One 7: e38544 10.1371/journal.pone.0038544 22685581 PMC3369835

[45] Hoffmann AA, Ross PA, Rasic G (2015) *Wolbachia* strains for disease control: ecological and evolutionary considerations. Evol Appl 8: 751–768. 10.1111/eva.12286 26366194 PMC4561566

[46] Frentiu FD, Robinson J, Young PR, McGraw EA, O'Neill SL (2010) *Wolbachia*-mediated resistance to Dengue virus infection and death at the cellular level. PLoS One 5: e13398 10.1371/journal.pone.0013398 20976219 PMC2955527

[47] Hussain M, Etebari K, Asgari S (2016) Functions of Small RNAs in Mosquitoes. Adv Insect Physiol 51: 189–222.

[48] Mayoral JG, Etebari K, Hussain M, Khromykh AA, Asgari S (2014) *Wolbachia* infection modifies the profile, shuttling and structure of microRNAs in a mosquito cell line. PLoS One 9: e96107 10.1371/journal.pone.0096107 24759922 PMC3997519

[49] Carpenter S, Aiello D, Atianand MK, Ricci EP, Gandhi P, et al (2013) A long noncoding RNA mediates both activation and repression of immune response genes. Science 341: 789–792. 10.1126/science.1240925 23907535 PMC4376668

[50] Peng X, Gralinski L, Armour CD, Ferris MT, Thomas MJ, et al (2010) Unique signatures of long noncoding RNA expression in response to virus infection and altered innate immune signaling. Mbio 1 (5). 10.1128/mBio.00206-10 PMC2962437 20978541

[51] Jayakodi M, Jung JW, Park D, Ahn Y-J, Lee S-C, et al (2015) Genome-wide characterization of long intergenic non-coding RNAs (lincRNAs) provides new insight into viral diseases in honey bees *Apis cerana* and *Apis mellifera*. BMC Genomics 16: 680 10.1186/s12864-015-1868-7 26341079 PMC4559890

[52] Atianand MK, Fitzgerald KA (2014) Long non-coding RNAs and control of gene expression in the immune system. Trends Mol Med 20: 623–631. 10.1016/j.molmed.2014.09.002 25262537 PMC4252818

[53] Ulitsky I, Bartel DP (2013) lincRNAs: Genomics, Evolution, and Mechanisms. Cell 154: 26–46. 10.1016/j.cell.2013.06.020 23827673 PMC3924787

[54] Struhl K (2007) Transcriptional noise and the fidelity of initiation by RNA polymerase II. Nat Struct Mol Biol 14: 103–105. 10.1038/nsmb0207-103 17277804

[55] Barriocanal M, Camero E, Segura V, Fortes P (2015) Long non-coding RNA BST2/BISPR is induced by IFN and regulates the expression of the antiviral factor tetherin. Front Immunol 5: 655 10.3389/fimmu.2014.00655 25620967 PMC4288319

[56] Zhao H, Chen M, Lind SB, Pettersson U (2016) Distinct temporal changes in host cell lncRNA expression during the course of an adenovirus infection. Virology 492: 242–250. 10.1016/j.virol.2016.02.017 27003248 PMC7111612

[57] Watanabe T, Cheng EC, Zhong M, Lin HF (2015) Retrotransposons and pseudogenes regulate mRNAs and IncRNAs via the piRNA pathway in the germline. Genome Res 25: 368–380. 10.1101/gr.180802.114 25480952 PMC4352877

[58] Roberts TC, Morris KV, Weinberg MS (2014) Perspectives on the mechanism of transcriptional regulation by long non-coding RNAs. Epigenetics 9: 13–20. 10.4161/epi.26700 24149621 PMC3928176

