# Supplementary figures and images for: Identification of Aedes aegypti Long Intergenic Non-coding RNAs and Their Association with Wolbachia and Dengue Virus Infection
Source: PLoS Negl Trop Dis. 2016 Oct 19;10(10):e0005069. doi: 10.1371/journal.pntd.0005069 (PMC5070814; doi:10.1371/journal.pntd.0005069)

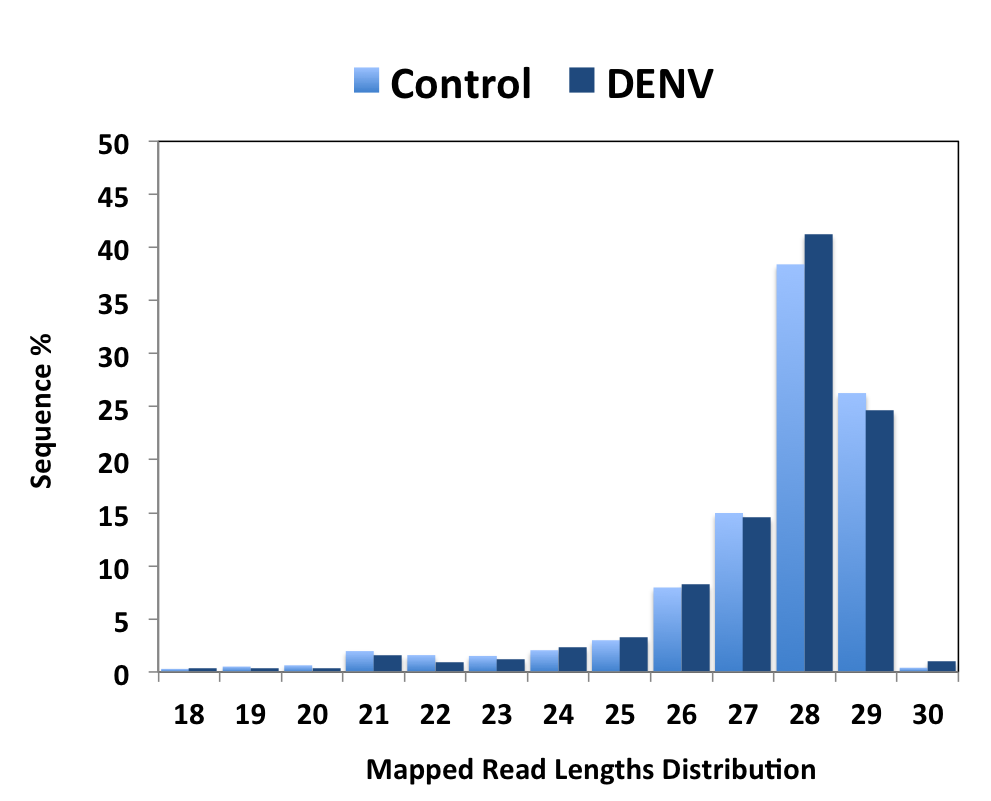

Supplement: S1 Fig — (TIF) [file pntd.0005069.s001.tif]

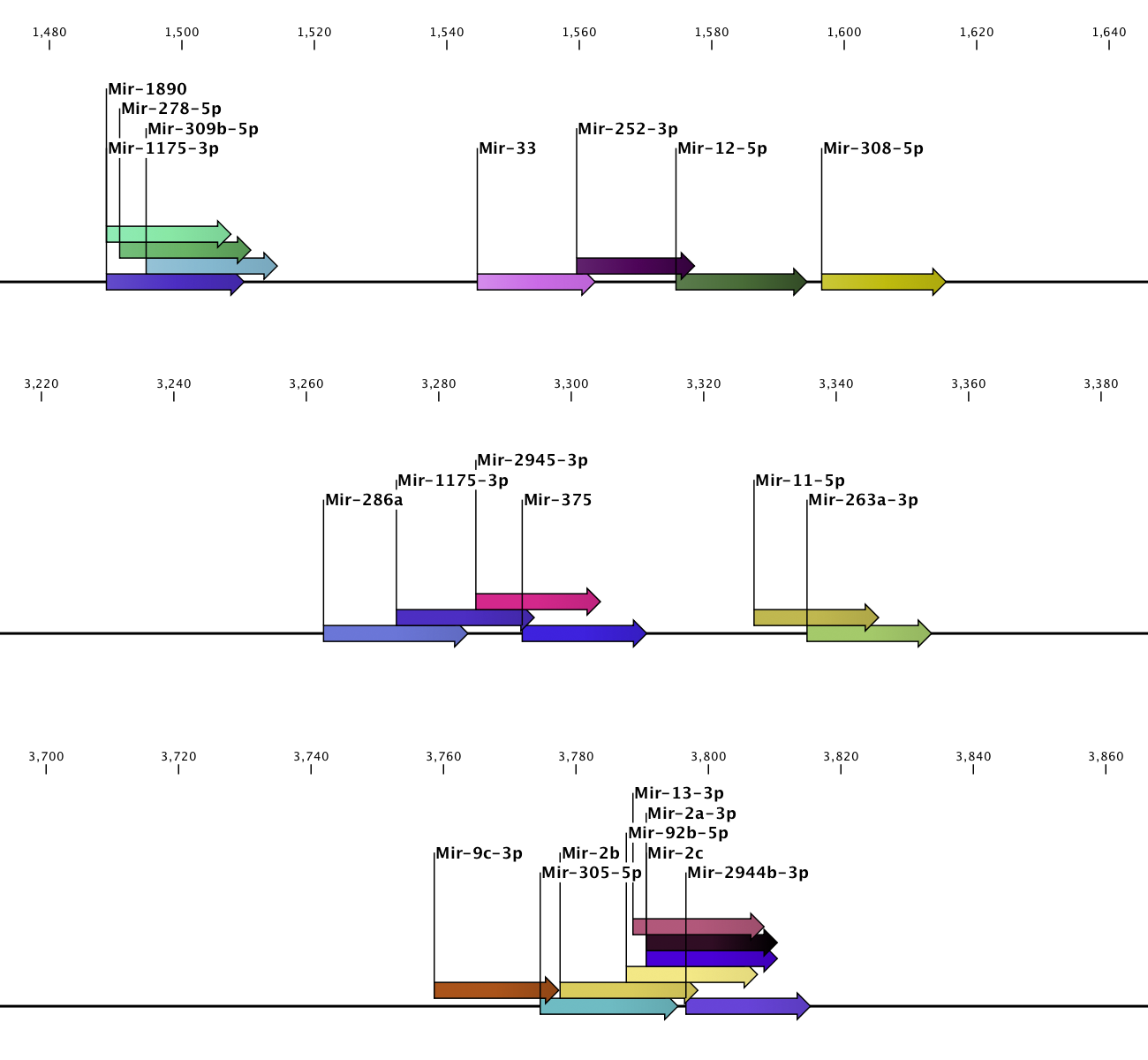

Supplement: S2 Fig — (TIF) [file pntd.0005069.s002.tif]
