# Supplementary material for: Identification of Aedes aegypti Long Intergenic Non-coding RNAs and Their Association with Wolbachia and Dengue Virus Infection
Source: PLoS Negl Trop Dis. 2016 Oct 19;10(10):e0005069. doi: 10.1371/journal.pntd.0005069 (PMC5070814; doi:10.1371/journal.pntd.0005069)
Supplement: S1 Table — (DOCX) [file pntd.0005069.s003.docx]

**Table S1. List of primers used in this study.**

| **Primer** | **Sequence** |
| --- | --- |
| lincRNA_1317 (Forward) | CACGAGCACTGGCTTTGAAC |
| lincRNA_1317 (Reverse) | TCACATAACTGCTTCGCCGT |
| lincRNA_2329 (Forward) | AATGGTACGTCGCAATAGGC |
| lincRNA_2329 (Reverse) | TCCGAACGAGAAGGAGAATG |
| lincRNA_2782 (Forward) | GCTTTTGCTGCTCACTTTCC |
| lincRNA_2782 (Reverse) | ACGTTGAACGACGATGACTG |
| lincRNA_1613 (Forward) | CGAAAACGCAAACCCCATCC |
| lincRNA_1613 (Reverse) | ACTGCATTCGTCCGGTAGTC |
| lincRNA_1879 (Forward) | CACCGATACAAATCGCACAC |
| lincRNA_1879 (Reverse) | TTATCGGTCATGGTGTCACG |
| lincRNA_1943 (Forward) | ATCAGAATCTGCGGTGGAAG |
| lincRNA_1943 (Reverse) | CACACCAGATGGTGCAATTC |
| lincRNA_2016 (Forward) | GCAATAGATTTGGCGACCTC |
| lincRNA_2016 (Reverse) | AGCCAATAAGCCTTCACAGC |
| lincRNA_2329 RNAi (Forward) | TAATACGACTCACTATAGGGCATTGAATCCAGTTTCACGA |
| lincRNA_2329 RNAi (Reverse) | TAATACGACTCACTATAGGGGCGCGACCGTAGAACCTAA |
| lincRNA_1613 RNAi (Forward) | TAATACGACTCACTATAGGGCGCAGTGGAGGAAACGAC |
| lincRNA_1613 RNAi (Reverse) | TAATACGACTCACTATAGGGCAGCTTCCACGCATCTCC |
| lincRNA_1317 RNAi (Forward) | TAATACGACTCACTATAGGGCGTAGTCTAGCGCAGGGATG |
| lincRNA_1317 RNAi (Reverse) | TAATACGACTCACTATAGGGACTGACGTCAAGTCACGCAT |
| DENV-NS2A-For | GGTATGGTGGGCGCTACTA |
| DENV-NS2A-Rev | CAAGGCTAACGCATCAGTCA |
| RPS17 (Forward) | CACTCCCAGGTCCGTGGTAT |
| RPS17 (Reverse) | GGACACTTCCGGCACGTAGT |
